# Compressed Sensing mm-Wave SAR for Non-Destructive Testing Applications Using Multiple Weighted Side Information

**DOI:** 10.3390/s18061761

**Published:** 2018-05-31

**Authors:** Mathias Becquaert, Edison Cristofani, Huynh Van Luong, Marijke Vandewal, Johan Stiens, Nikos Deligiannis

**Affiliations:** 1 CISS Department, Royal Military Academy, 30 Av. de la Renaissance, B-1000 Brussels, Belgium; edison.cristofani@elec.rma.ac.be (E.C.); marijke.vandewal@rma.ac.be (M.V.); 2 ETRO Department, Vrije Universiteit Brussel, Pleinlaan 2, B-1050 Brussels, Belgium; hvanluon@etrovub.be or huynh.luong@fau.de (H.V.L.); jstiens@etrovub.be (J.S.); ndeligia@etrovub.be (N.D.); 3 IMEC, Kapeldreef 75, 3001 Leuven, Belgium

**Keywords:** compressed sensing, synthetic aperture radar, mm-wave sensing, side information, non-destructive testing, additive manufacturing

## Abstract

This work explores an innovative strategy for increasing the efficiency of compressed sensing applied on mm-wave SAR sensing using multiple weighted side information. The approach is tested on synthetic and on real non-destructive testing measurements performed on a 3D-printed object with defects while taking advantage of multiple previous SAR images of the object with different degrees of similarity. The tested algorithm attributes autonomously weights to the side information at two levels: (1) between the components inside the side information and (2) between the different side information. The reconstruction is thereby almost immune to poor quality side information while exploiting the relevant components hidden inside the added side information. The presented results prove that, in contrast to common compressed sensing, good SAR image reconstruction is achieved at subsampling rates far below the Nyquist rate. Moreover, the algorithm is shown to be much more robust for low quality side information compared to coherent background subtraction.

## 1. Introduction

Terahertz and mm-wave sensors have the advantages of using harmless radiation, as opposed to X-ray scans. This type of sensor is able to image the inner structure of the objects from a stand-off distance, in contrast to ultrasound NDT, where contact with smooth surfaces is required [1,2]. The sensor used in this work is a mm-wave Synthetic Aperture Radar (SAR), delivering high resolution in-depth images of the inner structure of an object. Two configurations are possible: in a SAR configuration, the radar antenna moves along the object, creating synthetically a large antenna and emitting wideband frequency modulated signals; or the object can be placed on a moving stage and be passed in front of the sensor in an Inverse SAR (ISAR) configuration. Due to the mechanical scanning, the 3D-inspection of large objects becomes time consuming and thereby impacts the off-duty time of the equipment. The SAR sensing process produces high data volumes, inconvenient for data handling or transfer and requiring demanding storage capabilities [3].

Over the past decade, Compressed Sensing (CS) has been applied to a large variety of applications challenged by large data volumes, incomplete data, long measurement times or expensive equipment, such as: medical imaging [4], telecommunications [5] or radar imaging [6]. The CS performance for reconstructing an unknown signal from a small number of measurements largely depends on the sparsity or compressibility of that signal; a requirement that is rarely satisfied in many SAR applications [7], leading to mediocre reconstruction performances. If besides the prior assumption of sparseness, other prior information is available, such as a model of the object or a prior measurement, e.g., recursive inspections for structural health monitoring, this side information should be exploited to further decrease the number of measurements needed to obtain the required information and potentially achieve greatly reduced measurement times and data volumes. A fast-forward solution and alternative for our strategy, for inserting the prior information into the reconstruction algorithm, is background subtraction [8], used for example for MR imaging [9] and in through-the-wall radar imaging [10]. Recently, a variety of CS reconstruction techniques with prior information has been explored, integrating the prior information alongside the sparsity assumption into the CS algorithm [11,12,13,14]. The prior information can contain multiple side information [15,16,17] or even heterogeneous side information [18].

The chosen approach for adding prior information to the CS algorithm is tested on real data obtained from NDT measurements on a 3D-printed test object with artificially added anomalies. Additive Manufacturing (AM) has become an extremely popular technology and is widely used for prototyping, copying and producing objects [19]. 3D-printing allows for in situ fabrication of objects manufactured from metallic, ceramic, polymeric materials or a combination, with complex and optimized geometries. Many parameters, for example, high temperature gradients, calibration problems or unadapted printing speeds, can induce weaknesses or defects inside the objects. Performant Non-Destructive Testing and Evaluation (NDT&E) techniques for AM applications are highly needed in order to assure the integrity of the printed object [20,21,22], e.g., for critical structures used in the aviation or the space industry [23].

### 1.1. Contributions

In this work, an innovative Compressed Sensing (CS) algorithm incorporating autonomously weighted Side Information (SI) is proposed for mm-wave SAR measurements. The contributions can be summarized as follows:

1. To the best of the authors knowledge, CS with weighted SI is applied on SAR measurements for the first time in this work.
2. Extensive simulations are performed in order to evaluate the applicability of the proposed technique, the reconstruction performance when subsampling, the robustness against poor quality SI and against the use of multiple SIs with different qualities. The results are compared with common CS reconstruction and background subtraction.
3. The results obtained through the synthetic measurements are confirmed with experiments on real mm-wave NDT measurements of a 3D-printed test object, proving its direct applicability and demonstrating its high performance level.

### 1.2. Organization

The remainder of this paper is organized as follows. Section 2 describes the notions of mm-wave SAR, CS and CS with Side Information (SI) which are applied in the later sections of this paper. Section 3 starts with the description of the measurement setup, used for the synthetic experiments, and tests the reconstruction performance and robustness of the proposed solution. The conclusions drawn from the simulations in Section 3 are then confirmed by a series of experiments on real NDT mm-wave SAR measurements. Section 4, finally, concludes the work and gives some hints for future work.

## 2. Compressive Sensing mm-Wave SAR with Side Information

### 2.1. mm-Wave SAR

**Figure 1:**
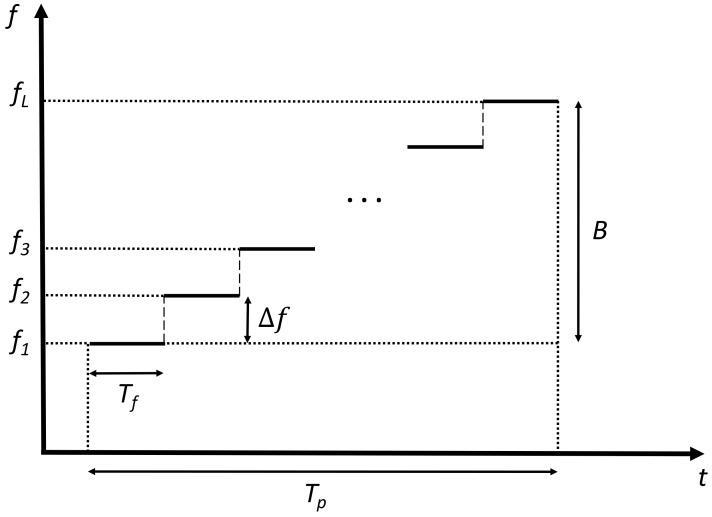
Stepped-frequency continuous wave signal.

The mm-wave sensor used for the experiments described in this document is a Vector Network Analyzer (VNA) emitting *L* discrete frequencies $f_{l}$, evenly spaced (with frequency step = $\Delta_{f}$) over the total bandwidth *B*. The emitted signal is thus a Stepped-Frequency Continuous Wave (SFCW) signal described by:

(1) $$s\left(t\right)=\sum_{l=1}^{L}\mathrm{rect}\left(\frac{t-lT_{p}}{T_{p}}\right)\exp\left(j2\pi f_{l}t\right),$$

where rect(.) is the rectangular function and $T_{p}$ the period for emitting the total bandwidth *B* (Figure 1).

The received signal by the sensor is the sum of the *K* signals backscattered on *K* reflectors characterized by reflection coefficients $a_{k,l}$ and located at distances $R_{k}$ from the sensor antenna. The reflected signal from scatterer *k* reaches the sensor antenna at t= the Round Trip Time (RTT), which is equal to $\frac{2R_{k}}{c}$, with *c* the propagation speed of the electromagnetic waves through the medium ($c=\frac{c_{0}}{\sqrt{\mu \epsilon }}$):

(2) $$s\left(t\right)=\sum_{k=1}^{K}\sum_{l=1}^{L}a_{k,l}\exp\left\{j2\pi f_{l}\left(t-RTT_{k}\right)\right\}.$$

The received signal after homodyne demodulation becomes:

(3) $$s_{b}\left(t\right)=\sum_{k=1}^{K}\sum_{l=1}^{L}a_{k,l}\exp\left\{-j2\pi f_{l}\frac{2R_{k}}{c}\right\}.$$

The range resolution obtained with this sensor is defined by the total emitted bandwidth and equal to:

(4) $$\Delta r_{ra}=\frac{c}{2B}.$$

In order to obtain a cross-range resolution of the same order of magnitude as the resolution in the range direction, a synthetic antenna aperture is created by moving the sensor in the cross-range direction. At each sensor position along the cross-range track, the beat signal (3) is measured and added to the growing raw-data matrix, which finally reaches dimensions equal to the number of sensor positions times the number of emitted frequencies (*L*). The high-resolution image is then obtained through the application of a SAR compression algorithm [24]. Alternatively, the SAR image can also be constructed by means of a Compressed Sensing (CS) algorithm [25].

### 2.2. Compressed Sensing

Consider an unknown signal $x\in C^{N}$ with at most k<<N nonzero entries, i.e., *x* is said to be *k*-sparse. Suppose further that we sense this unknown vector *x* by applying a set of n<N linear measurements, resulting in a measurement vector $y\in C^{n}$:

(5) y=Ax,

where $A\in C^{n\times N}$ is the observation matrix. In the context of SAR measurements: *x* is a vector describing the reflection characteristics of the discretized scene; the matrix *A* models the linear sensor by mapping the reflection characteristics of the scene into a vector *y*, containing the obtained beat signals. CS theory states that *x* can be reconstructed from this underdetermined set of equations with high probability if *A* satisfies the Restricted Isometry Property (RIP) [26,27] or a null-space property [28], and the estimation $\hat{x}$ for *x* can be found by solving the following Basis Pursuit (BP) problem:

(6) $$\min{∥x∥}_{1}\;s.t.\;y=Ax,$$

where ${∥x∥}_{p}:={\left(\sum_{i=1}^{n}\left|x_{i}\right|\right)}^{1/p}$ denotes the $\ell_{p}$-norm of *x*. For noisy measurements, y=Ax no longer holds and an accuracy threshold ϵ is introduced, resulting in the Basis Pursuit Denoising (BPDN) problem:

(7) $$\min{∥x∥}_{1}\;s.t.\;{∥Ax-y∥}_{2}\leq \epsilon .$$

In the literature, a number of recovery algorithms have been proposed, which can be classified into the following categories: greedy algorithms, convex and relaxation algorithms and Bayesian-based algorithms [29]. For this application, a high reconstruction quality performance is needed. For this reason, we opt for a basis pursuit algorithm.

### 2.3. CS with Side Information

The cornerstone of CS is the assumption that *x* is sparse or has a sparse representation in a known basis. By adding this prior knowledge to the set of underdetermined equations, the infinite set of candidates for $\hat{x}$ can be reduced to the single and correct solution *x*. Often, the sparsity of *x* is not the only knowledge available prior to the measurement. Additional information, commonly named as Side Information (SI), can be added to the reconstruction algorithm to further lowering the subsampling bound, ensuring the correct reconstruction of *x*. In this subsection, three strategies are explained for injecting this SI into the minimization Equation (7).

#### 2.3.1. Coherent Background Subtraction

Consider $z\in C^{N}$ to be a vector sharing a high degree of similarity with *x*. *z* can for example be a previous measurement of the object under test or a measurement of a similar object. This SI is equal to the unknown vector, except in a small number of its entries, and can be considered to be the background for the vector *x*. Minimizing the foreground, i.e., the vector (x−z), instead of minimizing the $\ell_{1}$-norm of *x*, will be more effective at low sampling rates since we can assume that ${∥x-z∥}_{1}<k$. The minimization Equation (7) becomes:

(8) $$\min{∥x-z∥}_{1}\;s.t.\;{∥A(x-z)-(y-Az)∥}_{2}\leq \epsilon ,$$

where the foreground is equal to (x−z) and with (y−Az) is the coherent subtraction of the synthetic measurements of the SI (Az) from the effective measurements *y*.

#### 2.3.2. $\ell_{1}\ell_{1}$-Minimization

Coherent background subtraction neglects the sparsity of *x* and concentrates on minimizing the number of nonzero elements of the foreground (x−z). A different approach for adding the SI *z* into the minimization equation is to minimize the weighted sum of the $\ell_{1}$ norm of *x* and the difference between *x* and *z* [12,30]:

(9) $$\min\left\{{∥x∥}_{1}+\beta g\left(x-z\right)\right\}\;s.t.\;{∥Ax-y∥}_{2}\leq \epsilon ,$$

with g(.) a function expressing the similarity between *x* and *z* and β>0 a trade-off factor between the sparsity of *x* and the similarity between *x* and *z*. Popular choices for g(.) in the literature are the $\ell_{2}$- and $\ell_{1}$-norm, resulting in $\ell_{1}\ell_{2}$- minimization and $\ell_{1}\ell_{1}$-minimization, respectively. The work in [31] compares both approaches and concludes that $\ell_{1}\ell_{1}$-minimization outperforms the $\ell_{1}\ell_{2}$ approach, and the authors further prove that β=1 delivers the sharpest undersampling bound:

(10) $$\min\left\{{∥x∥}_{1}+{∥x-z∥}_{1}\right\}\;s.t.\;{∥Ax-y∥}_{2}\leq \epsilon .$$

#### 2.3.3. Weighted $n\ell_{1}$-Minimization:

Suppose that we dispose over multiple SIs: $z_{j}$, with varying and a priori unknown qualities. From the different SIs, a mean SI: ${\mathrm{mean}}_{j}\left(z_{j}\right)$ can be computed and inserted into the background subtraction approach (8) where *z* is replaced by: ${\mathrm{mean}}_{j}\left(z_{j}\right)$. Alternatively, the $\ell_{1}\ell_{1}$-minimization problem (10) can be extrapolated towards the $n-\ell_{1}$-minimization equation:

(11) $$\min_{x}\left\{\frac{1}{2}{∥Ax-y∥}_{2}^{2}+\lambda \sum_{j=0}^{J}{∥x-z_{j}∥}_{1}\right\},$$

where $z_{0}=0$. These two approaches attribute the same impact to each of the SIs, neglecting possible differences in quality. SI of poor quality will thereby contaminate the mean SI or corrupt the minimization of (11). Alternatively, the work in [15] proposes a weighted multiple SI algorithm, RAMSIA, for solving the following minimization equation:

(12) $$\min_{x}\left\{\frac{1}{2}{∥Ax-y∥}_{2}^{2}+\lambda \sum_{j=0}^{J}\beta_{j}\left({∥W_{j}\left(x-z_{j}\right)∥}_{1}\right)\right\},$$

where $\beta_{j}\geq 0$ are the weights among the SIs and $W_{j}\geq 0$ is the diagonal weight matrix attributed to SI $z_{j}$ and whose diagonal elements ($w_{j1},w_{j2},\ldots ,w_{jn}$) are populated with the weights between the different elements of $z_{j}$. We note that (12) is reduced to the unweighted Equation (11) when choosing $\beta_{j}=1$ and $W_{j}=I$ (where *I* is the unit matrix). The weights $\beta_{j}$ will valorize the global impact of the SIs possessing a high degree of similarity compared to *x* and suppress the poor SIs during the minimization process, while the weights $W_{ji}$ regulate the relative impact of the different elements inside a single SI $z_{j}$. The implementation of RAMSIA is based on a Fast Iterative Shrinkage-Thresholding Algorithm (FISTA) [32] and iteratively updates the SI weights in two stages: first an update for the intra-SI weights $W_{j}$ is computed, followed by the calculation of the inter-SI weights $\beta_{j}$. A more detailed explanation of the RAMSIA algorithm and the pseudo-code can be found in [15]. The user can choose from three different stopping criteria for ending the iterative sequence: (1) the maximum number of iterations; (2) a threshold for the relative variation of the objective function; (3) the minimum number of non-zero components of the estimate for *x*.

## 3. Results

### 3.1. Simulations

This section describes a series of synthetic experiments conducted in order to test the efficiency of the algorithm for sparse reconstruction with multiple weighted side information for subsampling, for poor quality SI and for multiple SI with varying qualities. The performance of RAMSIA is evaluated against the results obtained with common CS and with the coherent background subtraction approach.

#### 3.1.1. Setup

The synthetic measurements in this section are performed with a simulated sensor presenting similar sensor specifications as the real mm-wave SAR sensor used during the NDT measurements. The relevant specifications are listed in Table 1. The sensor is based on a Vector Network Analyzer (VNA), and the measured quantity is the complex reflection parameter S11:

(13) $$S_{11}\left(f_{p}\right)=\frac{S_{Rx}\left(f_{p}\right)}{S_{Tx}\left(f_{p}\right)},$$

where $S_{Rx}\left(f_{p}\right)$ and $S_{Tx}\left(f_{p}\right)$ are the complex amplitudes of the received signal and the transmitted signal at frequency $f_{p}$, respectively. The $S_{11}$ reflection parameter is equal to the received signal after homodyne demodulation (3). For the simulated measurement, these values are obtained by applying the measurement matrix *A* on a random *k*-sparse reflectivity function *x* populated with the reflectivity coefficients of all *N* pixels composing the sensed scene. The size of the pixels is defined by the range and cross-range resolutions of the sensor. The matrix *A* models the sensing procedure and is column-wise populated with the vectorized sensor responses to a reflectivity function containing a single reflective component. Each column corresponds thus to a vectorized version of a measurement of size $n_{sp}\times n_{f}$ with $n_{sp}$ the number of sensor positions and $n_{f}$ the number of emitted frequencies:

(14) $$A=\left[\begin{array}{cccccc} S_{11}^{x_{1,1}}\left(sp_{1},f_{1}\right) & S_{11}^{x_{1,2}}\left(sp_{1},f_{1}\right) & \ldots & S_{11}^{x_{2,1}}\left(sp_{1},f_{1}\right) & \ldots & S_{11}^{x_{n_{sp},n_{f}}}\left(sp_{1},f_{1}\right)\\ S_{11}^{x_{1,1}}\left(sp_{1},f_{2}\right) & S_{11}^{x_{1,2}}\left(sp_{1},f_{2}\right) & \ldots & S_{11}^{x_{2,1}}\left(sp_{1},f_{2}\right) & \ldots & S_{11}^{x_{n_{sp},n_{f}}}\left(sp_{1},f_{2}\right)\\ \vdots & \vdots & \ddots & \vdots & \ddots & \vdots \\ S_{11}^{x_{1,1}}\left(sp_{1},f_{n_{f}}\right) & S_{11}^{x_{1,2}}\left(sp_{1},f_{n_{f}}\right) & \ldots & S_{11}^{x_{2,1}}\left(sp_{1},f_{n_{f}}\right) & \ldots & S_{11}^{x_{n_{sp},n_{f}}}\left(sp_{1},f_{n_{f}}\right)\\ S_{11}^{x_{1,1}}\left(sp_{2},f_{1}\right) & S_{11}^{x_{1,2}}\left(sp_{2},f_{1}\right) & \ldots & S_{11}^{x_{2,1}}\left(sp_{2},f_{1}\right) & \ldots & S_{11}^{x_{n_{sp},n_{f}}}\left(sp_{2},f_{1}\right)\\ \vdots & \vdots & \ddots & \vdots & \ddots & \vdots \\ S_{11}^{x_{1,1}}\left(sp_{n_{sp}},f_{n_{f}}\right) & S_{11}^{x_{1,2}}\left(sp_{n_{sp}},f_{n_{f}}\right) & \ldots & S_{11}^{x_{2,1}}\left(sp_{n_{sp}},f_{n_{f}}\right) & \ldots & S_{11}^{x_{n_{sp},n_{f}}}\left(sp_{n_{sp}},f_{n_{f}}\right) \end{array}\right],$$

where $S_{11}^{x_{i,j}}\left(sp_{t},f_{p}\right)$ denotes the $S_{11}$ parameter measured at sensor position $sp_{t}$ and at frequency $f_{p}$ of a scene with a single nonzero scatterer located at position $x_{i,j}$ in the 2D-scene. In other words: $S_{11}^{x_{i,j}}\left(sp_{t},f_{p}\right)$ is the beat signal (3) with $f_{l}=f_{p}$, and $R_{k}$ is the distance between the scatterer at position $x_{i,j}$ and the sensor at position $sp_{t}$. Different strategies can be adopted to undersample the measurements: (1) random sampling of the frequencies over the whole SAR measurement; (2) random sampling of SAR sensor positions; (3) a combination of position and frequency sampling [33]. In this work, the authors chose to apply a pseudo-random frequency selection over the whole SAR measurement. This strategy corresponds with performing a synthetic measurement with *n* randomly selected rows of *A*.

#### 3.1.2. Robustness Against Subsampling

This experiment evaluates the performances of the coherent background subtraction approach and the RAMSIA algorithm for the reconstruction of a random *k*-sparse vector (k=10) of length N=200 compared against common compressed sensing. The reconstruction quality is measured as the Euclidean distance between the original vector *x* and its reconstructed version $\hat{x}$, for an increasing number of samples. The similarity between the unknown vector *x* and the SI *z* is quantified as the fraction of elements with a different entry and named the change rate. For this experiment, the change rate is fixed to 5%. For each subsampling rate from 2% up to 60% (with a step of 2%), the reconstruction was executed 32 times with a different pseudo-random sampling scheme in order to render the results independent of the selection of samples.

**Figure 2:**
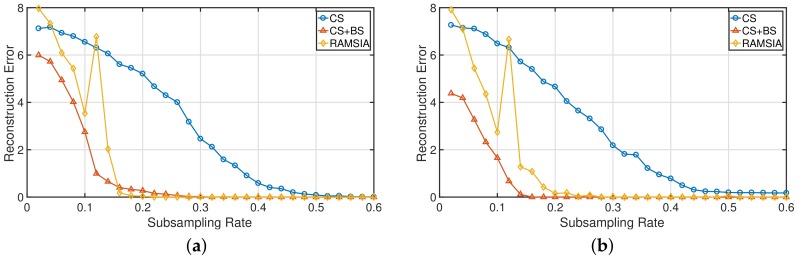
Mean reconstruction error (${∥x-\hat{x}∥}_{2}$) for increasing subsampling rates obtained with CS, CS with background subtraction and RAMSIA. (**a**) Sparsity(*x*) ≤ sparsity(*z*); (**b**) sparsity(*x*) ≥ sparsity(*z*).

The metric for evaluating the performances of the different algorithms is the Euclidean distance between the original vector and the recovered vector (${∥x-\hat{x}∥}_{2}$). Figure 2 depicts the reconstruction error obtained after reconstruction with a common BP algorithm (blue curve), with background subtraction (red curve) and with RAMSIA (yellow curve). Both for the results in (a) and in (b), the change rate was equal to 5%. In (a), *x* was first created with a sparsity equal to 5%, and thereafter, 10 randomly selected entries of *x* were given a different value in order to obtain *z*. This involves that *x* happens to be sparser than *z* in (a), while in (b), the inverse course of action for generating *x* and *z* was adopted, resulting in a sparser vector *z* compared to *x*.

The background subtraction reconstruction and the RAMSIA algorithm attain comparable reconstruction performances and clearly outperform common BP without SI. In the case that *x* is sparser than *z*, the RAMSIA algorithm reaches a reconstruction error equal to zero at a slightly lower subsampling rate than the background subtraction approach. In the inverse case (b), the background subtraction approach performs better than RAMSIA. This difference in reconstruction performance can be attributed to the fact that both reconstruction approaches minimize the $\ell_{1}$-norm of the difference between the two vectors, while only the algorithm with weighted SI minimizes the $\ell_{1}$-norm of *x* and will thus be affected by its sparsity.

#### 3.1.3. Robustness against Poor Quality SI

In order to test the impact of the quality of the SI on the reconstruction performance of both algorithms, a set of synthetic experiments was performed using vectors *z* with different qualities. The random vector *x* of length N=200 has again 10 nonzero random entries and is reconstructed using SI with change rates equal to 10%, 15%, 20%, 25%, 30% and 99%.

**Figure 3:**
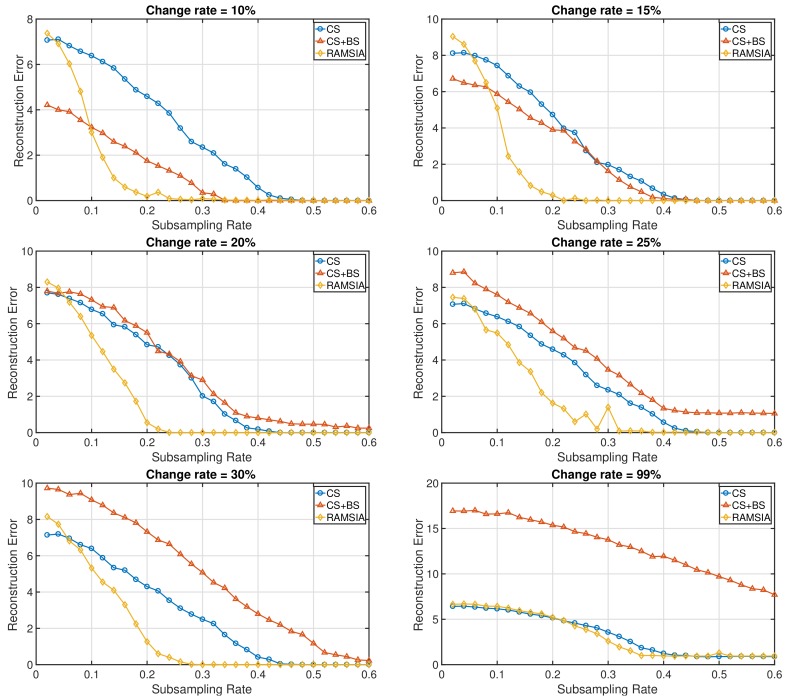
Mean reconstruction error (${∥x-\hat{x}∥}_{2}$) for increasing subsampling rates obtained with CS (blue curve), CS with background subtraction (red curve) and RAMSIA (yellow curve) using one Side Information (SI) with different quality levels (change rates: 10%, 15%, 20%, 25%, 30% and 99%).

The results in Figure 3 show that the RAMSIA algorithm outperforms the background subtraction algorithm when the quality of the SI decreases. At a change rate of 20%, the background subtraction approach performs at the same level as common BP, and the SI no longer has a positive impact; while the RAMSIA algorithm continues to filter out the relevant information hidden in the SI, resulting in much better reconstruction performances at low subsampling rates. If the quality of the SI further decreases, the SI pollutes the background subtraction reconstruction, resulting in even worse reconstruction performances. In the extreme case, when the SI has nothing in common with the original signal *x*, in this experiment simulated by choosing a change rate = 99%, a similar reconstruction performance is achieved for RAMSIA and common CS without SI. This illustrates the excellent performance of the adaptive weights used in RAMSIA.

#### 3.1.4. Multiple SI with Varying Qualities

In this section, different scenarios with multiple SIs are simulated. The setup is identical to the experiments described in the previous sections. For these experiments, *x* has a length of 200 and has 20 nonzero elements. Three different SIs are used: (1) SI1: a single SI, characterized by a change rate of 5%; (2) SI2: two SIs, with identical change rates of 5%; and (3) SI3: two SIs, with change rates equal to 5% and 20%. The reconstruction performance is compared with the CS reconstruction without using SI.

**Figure 4:**
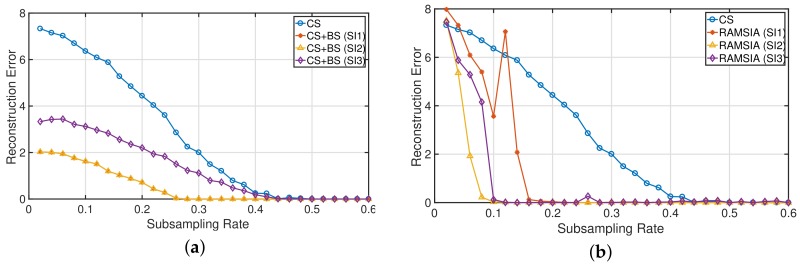
Mean reconstruction error (${∥x-\hat{x}∥}_{2}$) for increasing subsampling rates obtained with (**a**) CS (blue curve) and CS with background subtraction and (**b**) CS (blue curve) and RAMSIA for multiple SIs with varying change rates. (SI1) J = 1 (change rate 5%); (SI2) J = 2 (change rates = 5% and 5%); (SI3) J = 2 (change rates = 5% and 20%).

The results of this experiment obtained with the background subtraction approach are depicted in Figure 4a,b for the RAMSIA algorithm. RAMSIA outperforms the coherent background subtraction approach in all three scenarios. Adding side information of the same quality does not enhance the reconstruction performance in the case of coherent background subtraction: the curves obtained for SI1 and SI2 are perfectly identical. When using RAMSIA, the supplementary information is used, resulting in much better reconstruction performances. Adding SI information of poor quality to good quality SI has a negative impact on the background subtraction results, while the RAMSIA algorithm filters the relevant information out of the poor quality SI, which further enhances the reconstruction performance.

#### 3.1.5. Conclusions

The following conclusions can be drawn from the set of synthetic experiments described in this section:

1. Both CS with coherent background subtraction and CS with weighted SI enhance the robustness against severe subsampling and show comparable performances, dependent on the relative level of sparsity of the signal and the SI.
2. Background subtraction can jeopardize the reconstruction of a sparse vector if the SI is of poor quality, whereas the adaptive weighting algorithm is almost immune to low quality SI and can only have a positive impact on the reconstruction performance.
3. When multiple SIs are available, CS reconstruction using adaptive weighting of the SI filters out the relevant information of each SI and adds this complementary information to the reconstruction. Adding a new SI to the set of SIs cannot harm the reconstruction even if the SI has a low degree of similarity compared to the signal to be reconstructed.

### 3.2. Experiments on Real NDT Data

#### 3.2.1. Setup

**Figure 5:**
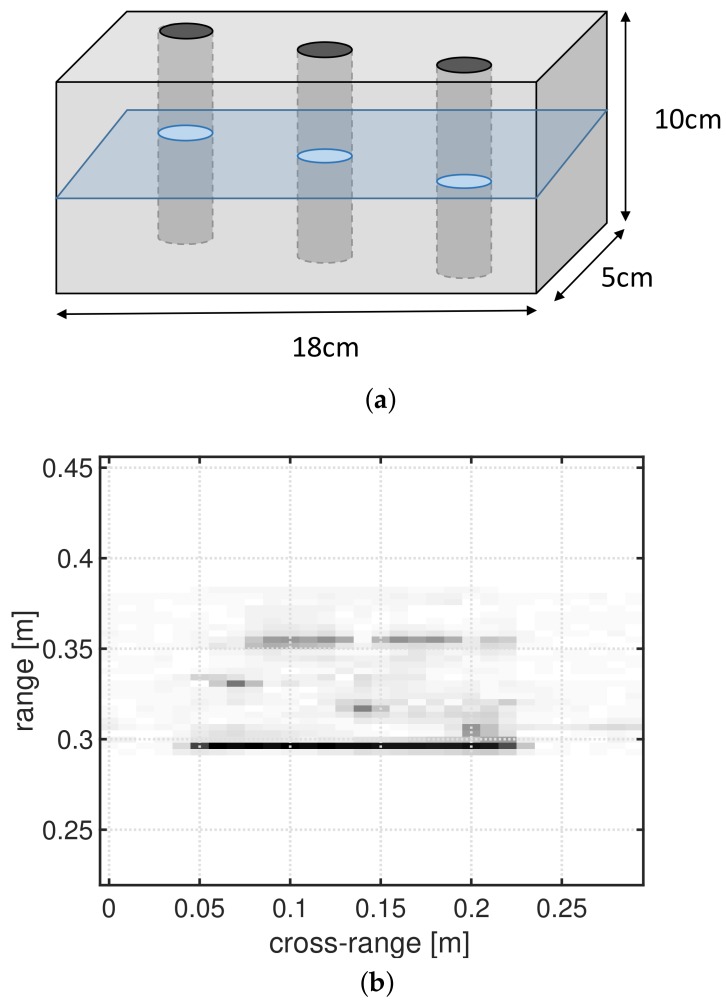
(**a**) Schematic representation of the test object (a massive 3D-printed ABS block, dimensions: 18 × 10 × 5 cm). Three defects were artificially added by drilling 3 identical vertical holes (radius: 5 mm) through the test object; (**b**) mm-Wave SAR image obtained from a full measurement of the test object with defects after applying a common range-Doppler algorithm. The image shows an in-depth view for a horizontal cut through the test object.

The sensor used for the NDT measurements described in this section has the same characteristics as listed in Table 1 and as the sensor used for the synthetic measurements. The real setup consists of a Vector Network Analyzer (mVNA, ABmm) connected to a single antenna and measuring the $S_{11}$ complex reflection parameter. The object under test (Figure 5a) is a 3D-printed rectangular block (dimensions: 18 × 5 × 10 cm made of ABS (Acrylonitrile-Butadiene-Styrene) polymer. The test object is placed on a scanning platform and moves over a total distance of 30 cm parallel to the opening of the antenna while taking measurements adhering to a stop and go method with steps of 1 cm. An in-depth image of a horizontal section through the object can be constructed from these measurements (Figure 5b).

**Figure 6:**
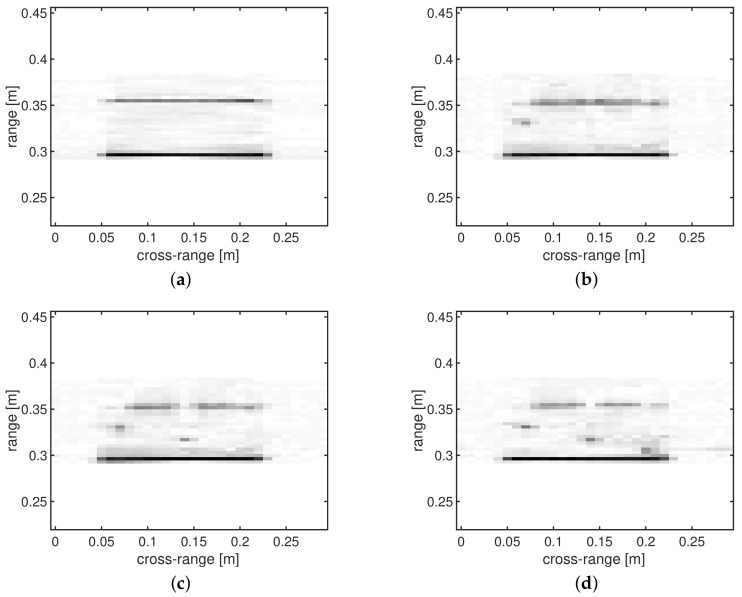
SAR image reconstructions from full data sampled at the Nyquist rate: (**a**) test object without defects (=$z_{1}$); (**b**) test object with one defect (=$z_{2}$); (**c**) test object with two defects (=$z_{3}$); and (**d**) test object with three defects (=*x*).

In total, four measurements are performed (Figure 6): the test object without any defects and three measurements of the test object with one, two and three defects at different ranges (the holes are drilled vertically through the test object and have a radius of 5 mm). For the experiments described in this section, the first three SAR images are considered to be three SIs, whereas the fourth measurement (the test object with three holes) is the unknown image that needs to be reconstructed. The Euclidean distances between the vectorized image that needs to be reconstructed (*x*) and the three SIs are equal to: 0.0805 for the test object without defects ($z_{1}$), 0.1071 for the test object with one defect ($z_{2}$) and 0.2408 for the test object with two defects ($z_{3}$). We note that the image of the test object without defects is surprisingly the most similar SI to *x*, whereas the test object with two holes is the SI with the lowest quality.

#### 3.2.2. Visual Evaluation

**Figure 7:**
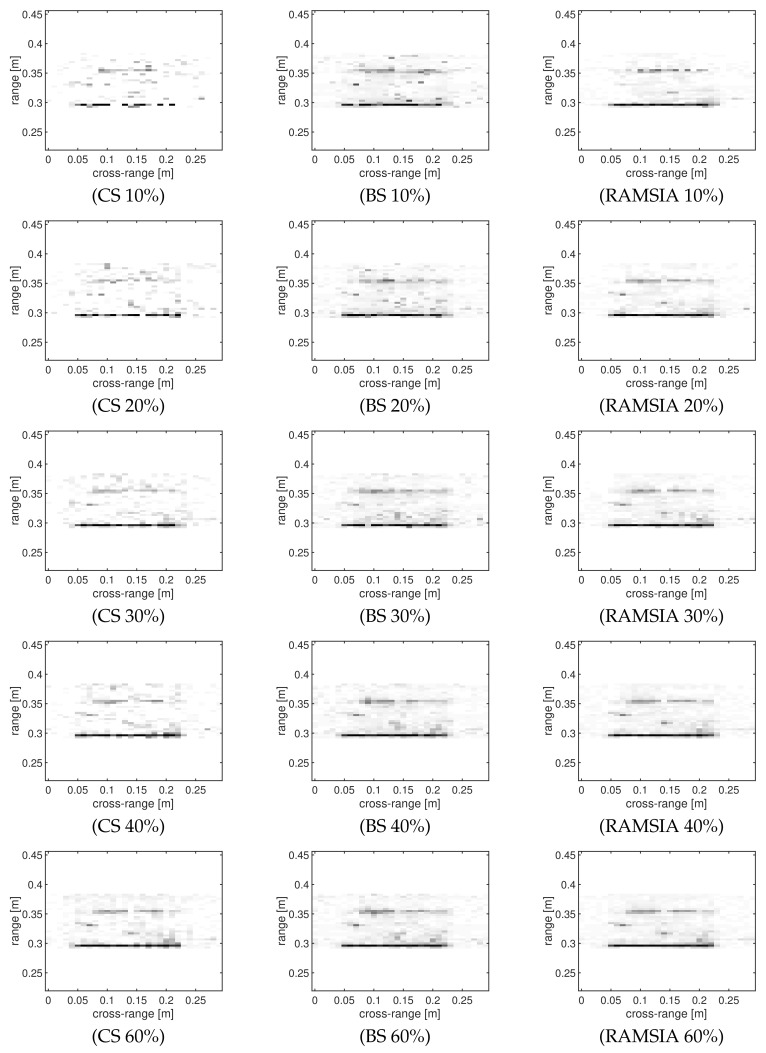
Visual comparison of the reconstruction performance of CS without SI (CS, first column), CS with background subtraction (BS, second column) and CS with weighted SI (RAMSIA, third column) for different subsampling rates: 10%, 20%, 30%, 40% and 60%.

Figure 7 shows a visual comparison for the reconstruction of the test object with three holes with SI = [$z_{1}z_{2}z_{3}$]. The first column shows the images obtained after the reconstruction with regular CS, whereas the second column shows the images obtained by coherent background subtraction, and the third column, finally, depicts the results obtained through the RAMSIA algorithm for different subsampling rates.

We can conclude from these images that the best reconstruction performance is achieved with RAMSIA: the defects are visible with only 20% of samples, whereas the defects are lost in the reconstruction clutter for the background subtraction algorithm and even not visible with common CS with 40% of samples.

#### 3.2.3. Robustness against Poor Quality SI

In order to confirm the conclusions drawn from the synthetic measurements and from the visual comparison, a series of experiments was conducted for several scenarios, using the real measurements and different combinations of the SIs.

**Figure 8:**
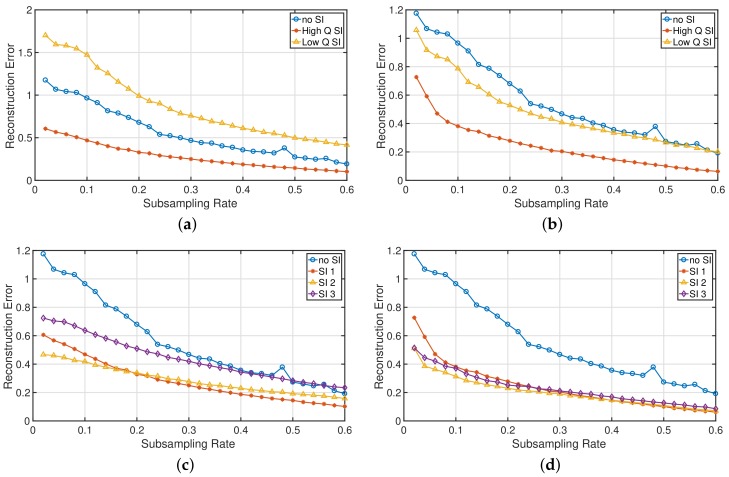
(**a**) Reconstruction error for increasing subsampling rates, obtained by CS with coherent background subtraction. In blue: CS without SI; in red: background subtraction with high quality SI ($z_{1}$); and in yellow: background subtraction with poor quality SI ($z_{2}$); (**b**) Reconstruction error for increasing subsampling rates, obtained by CS with weighted SI. In blue: CS without SI; in red: RAMSIA with high quality SI; and in yellow: RAMSIA with poor quality SI; (**c**) Reconstruction error for increasing subsampling rates, obtained by CS with coherent background subtraction. SI1 = [$z_{1}$], SI2 = [$z_{1}z_{2}$] and SI3 = [$z_{1}z_{2}z_{3}$]; (**d**) Reconstruction error for increasing subsampling rates, obtained by CS with weighted SI. SI1 = [$z_{1}$], SI2 = [$z_{1}z_{2}$] and SI3 = [$z_{1}z_{2}z_{3}$].

Figure 8a,b depicts the reconstruction error, expressed as Euclidean distance between *x* and the reconstruction $\hat{x}$, achieved by CS with background subtraction and by CS with weighted SI, respectively, using a single SI with two levels of quality. First, $z_{1}$, which shares a high degree of similarity with *x*, is added as SI (red curve), which decreases the reconstruction error significantly for both the background subtraction approach and the RAMSIA algorithm. Secondly, poor quality SI is added: $z_{3}$ (yellow curve). The reconstruction performance is slightly improved with the RAMSIA algorithm, while the background subtraction algorithm shows a strongly decreased performance compared to common CS without SI.

Figure 8c,d depicts the reconstruction errors for the SAR images obtained with background subtraction (c) and with RAMSIA (d), when adding multiple SIs: SI 1 = [$z_{1}$], SI 2 = [$z_{1}z_{2}$] and SI 3 = [$z_{1}z_{2}z_{3}$]. The conclusions drawn from the synthetic measurements are here confirmed: adding poor quality SI to the set of SIs contaminates the SI and deteriorates the reconstruction performance when applying background subtraction, whereas CS with weighted SI is almost not affected by adding a lower quality SI to the set of SIs.

#### 3.2.4. Adaptive Weights

**Figure 9:**
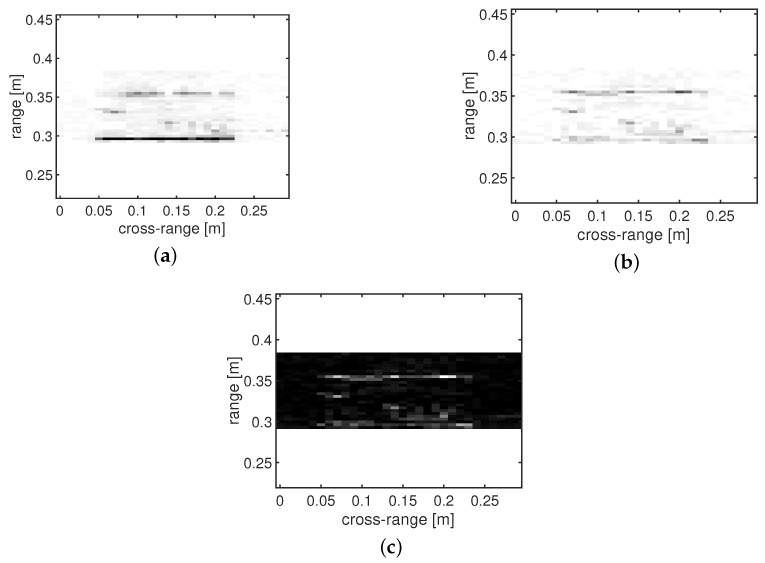
(**a**) Reconstructed SAR image with RAMSIA from 30% of the original number of samples with SI = $z_{1}$; (**b**) difference between *x* and $z_{1}$; (**c**) intra-SI weights $W_{1}$ positioned in the corresponding pixel.

**Figure 10:**
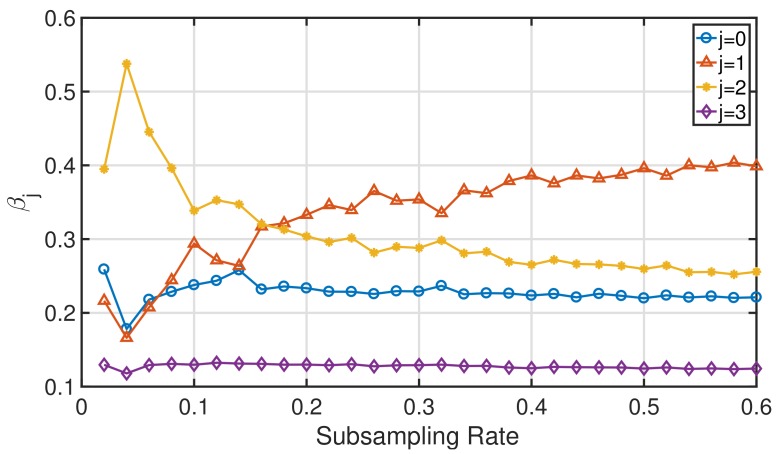
Distribution of the inter-SI weights $\beta_{j}$ for increasing subsampling rates.

The excellent performance of RAMSIA and its robustness against poor quality SI originate from the iterative adaptive weights. This is illustrated in Figure 9 and Figure 10.

Figure 9c shows the values of the elements on the diagonal of $W_{1}$ and placed on the corresponding positions in the image for a subsampling rate of 30%. The image in Figure 9a shows the SAR image obtained with RAMSIA at a subsampling rate of 30%, whereas Figure 9b depicts the difference between *x* and the SI $z_{1}$. The distribution of the weights attributed to the different pixels of $z_{j}$ clearly follows the result shown in Subfigure (b). The calculation of the intra SI-weights is thus successful even at low sampling rates.

Figure 10 shows the evolution of the inter-SI weights $\beta_{j}$ for increasing subsampling rates, attributed to the different SIs: $z_{1}$, $z_{2}$ and $z_{3}$. The order of the weights attributed to the three SIs corresponds to the order of the quality of the SIs, measured by calculating the Euclidean distance between the three SIs and *x*. From a subsampling rate less than 20%, the algorithm autonomously allocates the right amount of importance to each of the three SIs. Indeed, $z_{1}$ (the red curve) is the SI with the best quality, and the impact of this SI will be promoted; whereas $z_{3}$ (the purple curve) is the SI with the poorest quality and will almost be neglected during the reconstruction of *x*.

## 4. Conclusions

The idea for exploring the use of compressed sensing with multiple weighted side information applied to mm-wave SAR for NDT was motivated by two observations: (1) The sensor has to take a very large number of measurements in order to image the inner structure of an object of considerable dimensions, taking long measurement times and producing large amounts of data; (2) In many situations, the structure of the object under test is known through the modeling of the object or previous measurements. In a common setup, this information is not used, and the sensor invests time and power in sensing already known information.

In this paper, we proved the applicability of an innovative approach for CS exploiting multiple weighted SI applied on mm-wave SAR measurements. Through an extensive series of synthetic experiments, we compared the approach with common CS and background subtraction. These simulations led to the following conclusions: (1) CS with weighted SI outperforms common CS when the signal is reconstructed from a set of severely subsampled measurements; (2) whereas background subtraction can jeopardize the reconstruction of the unknown signal if the SI is of poor quality, our approach can only have a positive impact on the reconstruction of the signal, even in the extreme case that the SI has nothing in common with the signal; (3) when having access to multiple SIs, our approach will filter out the relevant information from the different SIs and allocate the right weight to each of the SIs in accordance with their quality.

These conclusions were then confirmed with tests on real NDT measurements, using a mm-wave SAR sensor for imaging the interior structure of a 3D-printed test-object. These tests prove the direct applicability and potential of the proposed solution.

References

## Figures and Tables

**Table 1:** Sensor characteristics of the simulated and real sensor used in the experiments.

NDT Sensor Parameter	Value
Starting frequency $f_{0}$	45 GHz
Bandwidth	30 GHz
Frequency step Δf	731.7 MHz
Scanning distance	0.30 m
Number of cross-range measurements	30
Number of frequencies	41
Aperture angle (−3 dB)	15°
